# Knowledge and Skills of Mothers/Care Givers of Children Under Five Years in Communities with Home Based Management of Malaria in Tamale, Northern Region, Ghana, 2013

**DOI:** 10.3934/publichealth.2016.4.923

**Published:** 2016-11-11

**Authors:** Mukaila Z. Mumuni, Mohammed A. Soghaier, Korkortiakor Baba S. Zankawah, Bukari Musah, Cynthia Kubio, Tanko Mahamadu, Assau Goodstaff

**Affiliations:** 1 Metropolitan Health Directorate, Ghana Health Service, Tamale, Ghana; 2 Epidemiologist, Directorate of Epidemiology & Zoonotic Diseases, Sudan Federal Ministry of Health, Khartoum, Sudan; 3 Public Health Specialist, Deputy Director, Claims Processing Center-Tamale National Health Insurance Authority, Ghana; 4 Public Health Specialist, Nanumba South District Health Directorate, Ghana Health Service, Wulensi; 5 Ghana Access and Affordability Programme

**Keywords:** Malaria in Ghana, Home based management, child mortality, child care givers

## Abstract

**Background:**

Malaria is still one of the major public health problems. More than 400 million cases of malaria are reported each year worldwide, Sub-Saharan Africa is the most affected region where about 90% of all malaria deaths in the world occur especially in children under five years of age. Home based management of Malaria showed a tremendous effect on reducing mortalities among children in Ghana.

**Objectives:**

to determine the current level of knowledge and skills of mothers in Tamale Metropolitan Area in the northern region of Ghana in terms of disease identification, management and transmission of malaria.

**Methodology:**

A cross sectional study conducted in 2013 involved 400 families and mothers/care givers with children less than five years were selected randomly and represented urban, peri-urbanand rural settings.

**Results:**

More than 90% of respondents identified malaria by presence of fever while 57.5% used fever as a cardinal sign. 91% of participants sought early treatment in urban and peri-urban settings while 85% did so in rural sites. 55% of participants administered the correct doses daily but only 17% of them knew the side effects of Antimalarial medications used. Almost all participants were aware about transmission of malaria, when to repeat the drug dose and usage of paracetamol as a medicine to reduce body temperature.

**Conclusion:**

The overall knowledge and skills demonstrated are encouraging, there is no much difference between urban and rural settings. Community based initiatives should be strengthened and promoted to provide homemade solutions to saving lives and resources.

## 1. Introduction

Till now Malaria is one of the major public health problems in the world. In sub Saharan Africa, It is estimated that 74% of its population live in highly endemic malaria areas and 19% in epidemic prone areas where about 90% of all malaria deaths in the world today take place especially in children under five years of age [1]. More than half of the deaths occur in rural areas mostly associated with intense malaria transmission [2]. The prompt and easy access to treatment is key to preventing severe forms of malaria and its fatalities among children under five years [3],[4].

Effective interventions including community based initiatives in Africa has led to dramatically reduced parasite transmission and saving lives [5]. In Ghana, malaria is hyper endemic with the entire population at risk. Transmission occur throughout the year with seasonal peak during rainy season especially in the northern part [6],[7]. Malaria is the leading cause of morbidity and mortality in Ghana accounting for about 38% of all out-patient illnesses, 36% of all admissions and 33% of all deaths in children below five years of age [8],[9]. It is estimated that between 3.1 and 3.5 million cases of malaria are reported in public health institutions each year.

Initiating early treatment which is one of the foundations of malaria control in sub-Saharan Africa depends upon early recognition of signs and symptoms at the home [10]. At this level individuals and caregivers recognize illness and decide on treatment options [11],[12]. In Africa, home management of illnesses is very important and popular. It is the first line of treatment for many diseases including malaria.

At home, mothers are primary caregivers and are usually the first to recognize signs of illness in their children. It has been demonstrated that educating mothers on malaria recognition and treatment can improve the effectiveness of malaria control programs [13]. Thus, the role mothers and care givers play in the prevention and treatment of malaria is very critical in its control strategies and successes. Consequently, information education and communication (IE&C) has been a major component of malaria control intervention strategies such as the home based management of malaria and community integrated management of childhood illnesses in Ghana. The foundation for HMM implementation is to provide caregivers with the most appropriate information that will enable them to recognize malaria, assess its severity, and take appropriate action [11],[12]. To improve access to antimalarial, the World Health Organization (WHO) promoted the home-based management of malaria (HMM) as a major strategy for Africa. HMM involves presumptively treating febrile children at or near home with antimalarial drugs distributed by trained members of the community [14]. Community distributors provide medications and educate primary caregivers about treatment of malaria, administration of antimalarial drugs, and recognition of severe illness. HMM helps to improve the knowledge of community members on malaria prevention as well as its early diagnosis, initiation and adherence to treatment. Access and availability to health infrastructure and social amenities vary drastically between urban and rural Ghana especially and this wanes as one move from urban to peri-urban and rural communities, hence, emphasizing the need for HMM to address this gap [12]. This study seeks to determine the current knowledge and skills of mothers and care givers on malaria in the three geographical distinct (urban, peri-urban and rural) settings through measuring their practice in malaria control and prevention.

Ethical approval was obtained from the Committee on Human Research, Publications and Ethics (CHRPE) of Kwame Nkrumah University of Science and Technology (KNUST) School of Medical Sciences; informed consent was obtained from parents of all study participants.

## 2. Materials and Methods

**Study design:** Cross sectional descriptive community based study.

**Study Area:** The study was carried out in Tamale Metropolis of the Northern Region of Ghana. The Metropolis has a total estimated land size of 646.90180 sqkm and population of 233,252 according to the 2010 Population and Housing Census. The Metropolis is divided into 4 sub district according to the health structure for easy administration.

**Study Duration:** The study took place from September to December 2013.

**Study population:** From families within areas that have been targeted by HMM, mothers/care givers with children under five years, the child should have had malaria within the past three months prior to the study, and mothers/care givers should have stayed for at least one year in their respective communities. Respondents who did not give consent or found not mentally prepared were excluded.

**Sampling technique:** A multi-stage cluster sampling technique was used for this study. The local health sector of Tamale Metropolis is administratively divided into four sub-districts. First, clusters and villages were randomly selected from each of the four sub-districts. Second, households were selected using systematic random sampling technique. Eligible mother/care giver per household was interviewed until the pre-determined number of respondents per that community was obtained. Where more than one eligible mother/care giver was present in a household, one of them was randomly selected using simple ballot. If there was no eligible mother/caretaker in the household, the next household was visited. In case the required number of eligible respondents was not obtained in a given community, the adjacent community originally not selected was visited to make up for the number required.

**Sample size:** Altogether, 400 participants were interviewed in the period from September to December 2013. The estimated proportion of knowledge about HMM in the district was assumed to be above 95% based on similar studies [15]. A design effect of 2 was used to adjust for cluster sampling. A rate of 20% for missed data and non-response are taken into account with assumed sampling error as ± 3%.

**Data collection and Processing:** Data was collected through structured questionnaire developed by the research team; the questionnaire was pre-tested and validated before the real data collection. It was self-administered questionnaire while illiterate mothers/care givers were assisted through trained data collectors.

## 3. Results

**Overall:** From the 400 participants, 140 (35%) were from urban setting while 135 (34%) and 125 (31%) were from peri-urban and rural settings respectively. The mean age of mothers/care givers was 25 years (±SD = 0.6). Majority (55.3%) of the respondents were less than 29 years and 40.8% were within the ages of 30–39 years. 89.3% were females and 89% were married. Only 6% had tertiary education while 8.3% had secondary education and 67% were traders or artisans. Table 1 summarizes the study participant's characteristics.

**Table 1.** The main individual characteristics of Study Participants.

Characteristics	Frequency (n)	Percent (%)
*Age*		
>29	221	55.3
30–39	162	40.5
40–49	15	4
50–70	2	1
*Gender of care giver*		
Male	43	10.8
Female	357	89.3
*Marital status of care giver*		
Married	356	89
Not married	44	11
*Level of education of care giver*		
Tertiary	24	6
Secondary	33	8.3
Basic	116	29
None	227	56.7
*Occupation of care giver*		
Farmer	57	14.3
Trader/Artisan	268	67
Government worker	36	9
Others	39	9.8

The overall malaria identification ability of respondents by the presence of fever was found to be 93.5%; however there was a variation in the location ranging from 97.1% (136) among the urban respondents, 91.1% (123) and 92% (115) among peri-urban and rural respondents respectively. Recognizing fever as the key indicator in identifying malaria among respondents was lower 230 (57.5%) compared to the general identification of malaria by fever presence. The knowledge gap varied from 65% (91) in the urban setup to 55.6% (75) in peri-urban and 51.2% (64) in the rural setting with a chi square statistic of 0.065 suggesting a weak relationship. Regarding early case detection, 75.5% (106) of the participants detected malaria within 24 hours in urban areas, whereas peri-urban and rural participants represented 86.7% (117) and 73.6% (92) respectively with statistical significant difference *p*-value = 0.020. Prompt seeking of care was more than 90% among urban and peri-urban respondents but 85% among the respondents without statistical difference (*p*-value = 0.28). More than 90% of respondents in the urban and peri-urban sought treatment promptly whiles 85% of the rural setting did so. Table 2 presents a summary of the specific findings among each study groups.

**Table 2.** Comparison of knowledge and skills between study participants from urban, peri-urban and rural settings.

Study variable	Yes n(%)	No n(%)	*P*-Value
*Malaria identification by presents of fever (Total)*	374(93.5)	26 (6.5)	0.091
Urban	136(97.1)	4 (2.9)	
Peri-Urban	123(91.1)	12 (8.9)	
Rural	115(92)	10 (8)	
*Using fever as the key feature in malaria (Total)*	230(57.5)	170 (42.5)	0.065
Urban	91(65)	49 (35)	
Peri-Urban	75(55.6)	60 (44.4)	
Rural	64(51.2)	61 (48.8)	
*Early case detection (within 24 hrs) (Total)*	315(79.8)	85 (20.2)	0.020
Urban	106(75.5)	34 (24.5)	
Peri-Urban	117(86.7)	18 (13.3)	
Rural	92(73.6)	33 (26.4)	
*Prompt seeking of care (within 24 hrs) (Total)*	357(89.2)	43 (10.8)	0.281
Urban	127(90.7)	13 (9.3)	
Peri-Urban	123(91.1)	12 (8.9)	
Rural	107(85.6)	18 (14.4)	
*Prompt initiating treatment (Total)*	346(86.5)	54 (13.5)	0.933
Urban	122(81.7)	18 (18.3)	
Peri-Urban	117(86.7)	18 (13.3)	
Rural	107(85.6)	18 (14.4)	
*Correct doses (2) in a day (Total)*	220(55)	180 (45)	0.224
Urban	84(60)	56 (40)	
Peri-Urban	67(49.6)	68 (50.4)	
Rural	69(55.2)	56 (44.8)	
*Correct duration of treatment (3 days) (Total)*	289(72.2)	111 (27.8)	0.02
Urban	92(65.7)	48 (34.3)	
Peri-Urban	109(80.7)	26 (19.3)	
Rural	88(70.4)	37 (29.6)	
*Awareness of side effects (Total)*	71(17.8)	329 (82.2)	<0.001
Urban	47(33.6)	93 (66.4)	
Peri-Urban	11(8.1)	124 (91.9)	
Rural	13(10.4)	112 (89.6)	
*Awareness of AA as drug of choice (Total)*	242(60.5)	158 (39.9)	0.391
Urban	81(57.9)	59 (42.1)	
Peri-Urban	88(65.2)	47 (34.8)	
Rural	73(58.4)	52 (41.6)	
*Correct duration to repeat a dose after vomiting (Total)*	295(73.8)	105 (26.3)	0.011
Urban	111(79.3)	29 (20.7)	
Peri-Urban	104(77)	31 (23)	
Rural	80(64)	45 (36)	
*Right time to take a dose after forgetting (Total)*	290(72.5)	110 (27.5)	0.171
Urban	104(74.3)	36 (25.7)	
Peri-Urban	103(76.3)	32 (23.7)	
Rural	83(66.4)	42 (33.6)	
*Use of paracetamol to reduce temperature (Total)*	314(78.5)	86 (21.5)	0.002
Urban	97(69.3)	43 (30.7)	
Peri-Urban	117(86.7)	18 (13.3)	
Rural	100(80)	25 (20)	
*Use of turbid sponging to reduce temperature (Total)*	251(62.8)	149 (37.2)	0.404
Urban	82(58.6)	58 (41.4)	
Peri-Urban	86(63.7)	49 (36.3)	
Rural	83(66.4)	42 (33.6)	
*Mosquito as a transmitter of malaria (Total)*	395(98.8)	5 (1.2)	0.018
Urban	135(96.4)	5 (3.6)	
Peri-Urban	135(100)	0 (0)	
Rural	125(100)	0 (0)	
**Urban N = 140,Peri-Urban = 135, Rural = 125, Total = 400**			

72.2% (289) of all the respondents gave treatment according to 3 days treatment duration with majority, 60% giving Artesunate-Amodiaquine. 60% (88) in urban, 49.6% (67) in peri-urban and 55.2% (69) in the rural area gave the correct dose of antimalarial drugs per day for complete course of 3 days.Only17% of the respondents knew antimalarial drugs have possible side effects with significant statistical difference between urban, peri-urban and rural settings (*p*-vale ≤ 0.001). More than 70% of the respondents repeated the doses at the right time when the child vomited after taking the malaria drugs. For reducing high body temperature among children who had malaria, 78.5% (314) use paracetamol while 62.8% (251) turpid sponging.

The knowledge of respondents on malaria transmission was 98.8%; 96.5% (135) in urban and 100% across peri-urban and rural settings with *p*-value = 0.018. The commonest source of information among the respondents was radio (local Fm stations) 30.7% (171) and health workers 26.9% (150) whereas impregnated treated bed nets (ITNs) was the widely used method of malaria prevention 35.5% (193).

## 4. Discussion

HMM implementation is to provide caregivers with the most appropriate information that will enable them to recognize malaria, assess its severity, and take appropriate and timely actions. The success of HMM therefore depend on effective information, education, and communication (IE&C) programmes[12]. Roll back malaria(RBM) target on access to information on HMM among mothers of children under five was 60% by 2005 and 80% by 2010 [16]. Experience has shown that the better the IE&C for communities, the better the program output regarding communicable diseases including malaria [17],[18], in this study, majority of the respondents across all the locations identified malaria by the presents of fever and as well used fever as the key feature in determining malaria in children, the same findings documented earlier in countries with similar conditions [19],[20]. However, this is regrettably not often the case especially if the wrong method or approach of IE&C is used [17]. Some underlying reasons that influence whether mothers/care givers sought treatment include uneasy access to health center, limited supply of affordable drugs including antimalarial drugs, perceived deficiencies in the performance of formal health services including poor clinical skills, attitude of health personnel and cultural beliefs [21]. In this and other studies, adherence to treatment was high among mothers and care givers across all the locations within the areas targeted by HMM [22]. Mothers/care givers mostly used Artesunate-Amodiaquine as first line of home treatment which is safe to undergo that purpose [23]. The failure of IE&C to emphasis on some salient components such as side effects of antimalarial drugs resulted in a larger proportion of respondents across all the locations not to know that this drug has side effects, this could have negative impact as some mother/care givers could stop administration of the drugs and this could lead to drug resistance and its associated problems. Similarly, mothers/care givers may prefer going in for other inappropriate antimalarial drugs or sought treatment elsewhere which stands the chance of increasing the number of severe malaria cases and fatalities [24]. This defeats one of the major goals of HMM strategy of empowering mothers/care givers to identify signs and symptoms of malaria early and appropriate treatment. In this study, the mostly used method to reduce high body temperature by mothers and care givers is the use of paracetamol, majority did so along with trupid sponging across all the locations, this is also observed from other studies [25]. This suggests that most mothers are able to properly manage elevated temperature among children under five years. In this study, almost all participants appropriately identified how malaria is transmitted as demonstrated by several studies [25]–[27]. It is estimated that about half of Ghanaians who leave in rural areas have absolutely no access to healthcare due to a lack of staff and inaccessible or non-existent roadways [28]. This indicates that there is an urgent need to expand HMM to allow such people to have access to information and education on disease prevention. Whereas this study finds no significant difference in the management of malaria among urban, peri-urban and rural respondents, other studies shows significant differences [29],[30] this could be due to difference of study populations or specific settings. In this current study, the most effective source of receiving malaria-related message was found to be the radio, while some other studies found the health workers as primary source of information there [19],[31].

## 5. Conclusion

In conclusion, the knowledge and skills of mothers/care givers in Tamale is encouraging, but this cannot be chiefly attributed to the implementation of HMM due to the study design used. To determine the impact of IE&C from HMM, further studies involving a more robust methodology is required. There is no difference of the effect of IE&C on the mothers/care givers ability to manage malaria in children less than five years in urban, peri-urban and rural Tamale. Knowledge of respondents on the side effects of Artesunate-Amodiaquine and other antimalarial drugs is poor in all the three geographical locations; this in addition to the duration of treatment should be the focus in subsequent IE&C activities.

## Abbreviations

*ACT*: Artemisinin Combination Therapy
*CHREP*: Committee on Human Research, Publications and Ethics
*HMM*: Home Base Management of Malaria
*IE&C*: Information Education and Communication
*ITNs*: Insecticide Treated Bed Nets
*KNUST*: Kwame Nkrumah University of Science and Technology
*RMB*: Roll Back Malaria
*WHO*: World Health Organization
